# Caveolin-1 enhances resveratrol-mediated cytotoxicity and transport in a hepatocellular carcinoma model

**DOI:** 10.1186/1479-5876-7-22

**Published:** 2009-03-25

**Authors:** Hui-ling Yang, Wei-qiong Chen, Xuan Cao, Andrea Worschech, Li-fen Du, Wei-yi Fang, Yang-yan Xu, David F Stroncek, Xin Li, Ena Wang, Francesco M Marincola

**Affiliations:** 1 Institute of Clinical Medicine, First Affiliated Hospital of University of South China, Hengyang, 421001, PR China; 2 Infectious Disease and Immunogenetics Section (IDIS), Department of Transfusion Medicine and Center for Human Immunology (CHI), National Institute of Health,10 Center Drive, Building 10, Bethesda, MD 20892, USA; 3 Institutes of Pharmacology and Pharmacy, University of South China, Hengyang, 421001, PR China; 4 Cancer Research Institute of Southern Medical University, Guangzhou 510515, PR China; 5 Genelux Corporation, San Diego Science Center, San Diego, California, USA; 6 Institute for Biochemistry, University of Würzburg, Am Hubland, Würzburg, Germany; 7 Cellular Processing Section, Department of Transfusion Medicine, National Institutes of Health, Bethesda, Maryland, USA

## Abstract

**Background:**

Resveratrol (RES), an estrogen analog, is considered as a potential cancer chemo-preventive agent. However, it remains unclear how RES is transported into cells. In this study, we observed that Caveolin-1(CAV1) expression can increase the cytotoxic and pro-apoptotic activity of RES in a dose- and time-dependent manner both *in vitro *and *in vivo *in a Hepatocellular Carcinoma animal model.

**Methods:**

High performance liquid chromatography (HPLC) demonstrated that RES intra-cellular concentration is increased about 2-fold in cells stably expressing CAV1 or CAVM1 (a scaffolding domain (81-101AA)-defective CAV1 mutant) compared to the untransduced human Hepatoblastoma cell line (HepG2) or after transduction with the green fluorescent protein (GFP) control vector. The increased intra-cellular transport of RES was abolished in cells stably expressing CAVM2 (a cholesterol shuttle domain (143-156AA)-defective CAV1 mutant) or CAVRNAi. In order to further characterize CAV1-dependent RES transport, we synthesized RES-dansyl chloride derivatives as fluorescent probes to visualize the transport process, which demonstrated a distribution consistent with that of CAV1 in HepG2 cells.

**Results:**

In addition, RES endocytosis was not mediated by estrogen receptor (ER) α and β, as suggested by lack of competitive inhibition by estrogen or Tamoxifen. Pathway analysis showed that RES can up-regulate the expression of endogenous CAV1; this activates further the MAPK pathway and caspase-3 expression.

**Discussion:**

This study provides novel insights about the role played by CAV1 in modulating cellular sensitivity to RES through enhancement of its internalization and trafficking.

## Background

Resveratrol (trans-3,4',5-trihydroxystilbene, RES), a phytoalexin found in grapes and other food products, is considered as a cardio-protective drug and a potential cancer chemo-preventive agent [1-6]. Through inhibitory effects on the oxidative modification of low density lipoproteins, RES can block internalization of oxidized lipoproteins responsible for its cardio-protective quality. In addition, RES can inhibit the growth of a variety of tumor cells *in vitro *and in animal models [7-9] through its anti-cancer properties including prevention, delay, and reversal of tumor initiation, promotion and progression. This is partly attributable to RES antioxidant activity and inhibitory effect on the hydroperoxidase activity of cyclooxygenase (Cox 1 and 2); furthermore, RES can inhibit transcription factors such as NF-kB, apoptotic protease activating factors (Apaf-1), and AHR, growth of estrogen responsive cells and induce accumulation of p53 [10-14]. Some studies indicated that RES has a molecular structure similar to diethylstilbesterol displaying estrogen-like agonistic and antagonistic activity. Therefore, RES could bind to the estrogen receptor (ER) and thereby activate the transcription of estrogen-responsive reporter genes [15-17]. However, most of the *in vivo *studies have failed to confirm the estrogen-like potential of RES.

Caveolins are plasma membrane rafts present in most cells, and were first characterized morphologically as small flask-shaped plasma membrane invaginations [18]. The typical caveolin-1 (CAV1) protein is a principal component of the caveolin family and its reduced or absent expression was shown in most human cancer cells. Several lines of evidence support CAV1 function as a "transformation suppressor" protein. Over expression of CAV1 blocks anchorage-independent growth of transformed cells. A varied array of functions has been proposed for caveolins, including modulation of signal transduction, endocytosis, potocytosis, and cholesterol trafficking. CAV1 can suppress epidermal growth factor tyrosine kinase (EGF), extra-cellular signal-regulated kinase (ERK), endothelial nitric-oxide synthase, threonine protein kinase, serine protein kinase such as Src family TK, PKCα, H-Ras via the CAV1 scaffolding domain that combines with these genes [19-24]. In addition, some reports suggest that CAV1 mediates mitogen-activated protein kinase (MAPK)-dependent CREB phosphorylation activating ERα and ERβ through its scaffolding domain similarly to ERα and ERβ activation by RES [15-17,25]. However, the CAV1-dependent mechanism(s) by which RES may trigger cell signaling remains to be determined.

This study analyzes whether and how CAV1 is involved in the cytotoxic and pro-apoptotic actions of RES in a human hepatocellular carcinoma (HCC) model. Lentiviral vectors expressing short hairpin RNAs (shRNAs) against the CAV1 gene [26] such as wild type (Wt CAV1), a scaffolding domain (81-101AA)-defective CAV1 mutant (CAVM1) and a cholesterol shuttle domain (143-156AA)-defective CAV1 mutant (CAVM2) were constructed and transfected into the human Hepatoblastoma cells HepG2; these cells display constitutively low levels of endogenous CAV1 [27,28]. The effects of WtCAV1, CAVM1 and CAVM2 expression on cell growth, apoptosis, and Topoisomerase-α -Topo II/P38 transcription in response to various doses (0~300 μm) of RES were analyzed *in vivo and in vitro*. Furthermore, the contribution of CAV1 to the influx and efflux of cellular RES was investigated by high performance liquid chromatography (HPLC) and its intracellular distribution by RES derivatives (RES-dansyl chloride) as fluorescent probes.

## Materials and methods

### Materials

Plasmid extraction kit (Promega, Madison, USA), and BCA protein quantitative kit (Pierce, Rockford, USA) were purchased. BlueRanger pre-dye protein molecule standard and protein fluorescence detection kit were from HyClone (South Logan, USA). Rabbit anti-human CAV-1(N-20), extra-cellular signal-related kinase1/2 (ERK1/2, K-23) and p38 kinase(H-147) polyclonal antibodies; mouse anti-human caspase-3(E-8), mouse anti-Topoisomerase-alpa (Ki-S1), mouse anti-human β-actin monoclonal antibody and phosphorylated proteins of ERK1/2(p- ERK1/2(E-4)); Cy3-conjugated goat anti-mouse IgG, goat anti-rabbit immunoglobulin G (IgG) and goat anti-mouse IgG antibodies coupled to horseradish peroxidase were all from Santa Cruz Biotechnology (Santa Cruz, USA). Alexa Fluor 488 -conjugated goat anti-rabbit IgG (H+L) antibody, Lipofectamine 2000 reagent, geneticin (G418) and blasticidin were purchased from Invitrogen (Carlsbad, USA); Cell medium and antibiotics were from Gibco-BRL (Paisley, Scotland, United Kingdom). Fetal bovine serum (FBS) was from HyClone (Logan, UT). Dansyl Chloride was purchased from Amresco. Resveratrol(trans-3,4',5-trihydroxystilbene, RES), special P38 mitogen-activated protein kinases inhibitor (SB203580), trans-Ferulic acid(trans-4-Hydroxy-3-methoxycinnamic acid, t-FA), Diethylstilbestrol(DES), Tamoxifen citrate and all other reagents used for immunofluorescence and Western blots were from Sigma and of the highest grade available.

### Plasmids

The mammalian GFP Fusion expression vector for human wild-type CAV1 was constructed by inserting the human CAV1 cDNA into pcDNA3.1/NT-GFP-TOPO [27]. Mutant CAV1 with the deletion of the scaffolding domain (CAVM1, CAV1-81-101aa) and mutant CAVM2 (lacking the lipid domain 143-156aa, CAVM2, CAV1-143-156) were generated by PCR mutagenesis using pcDNA3.1/NT-GFP-TOPO-CAV1 as a template and the GFP reporter vector as previously described. We used lentiviral expressed short hairpin RNAs (shRNAs) against CAV1.

### Cell culture

The human Hepatoblastoma carcinoma-2 HepG2 cell line was obtained from the Cell Bank, Chinese Academy of Sciences Shanghai Institute of Cell Biology, and cultured in Dulbecco's modified Eagle's medium supplemented with 10% fetal bovine serum (HyClone), 100 μg/ml penicillin and streptomycin, 4 mM/*L *glutamine, 1 mM MEM sodium pyruvate in a humidified 37°C incubator with 5% CO_2_. One day prior to the transfection, cells were plated into a 10 cm tissue culture plate and grown to 90%–95% confluence. The day after, 9 μg of plasmids (CAV1, CAVM1, CAVM2 and GFP reporter vector respectively) were transfected into the HepG2 cells using 10 μl of Lipofectamine 2000 reagent, according to the manufacturer's instructions. Forty-eight hours after transfection, Geneticin (500 μg/ml) was used to select stable transfectants. In addition, to obtain stable knockdown effect, the lentiviral supernatant expressed short hairpin RNAs (shRNAs) against the CAV1 gene was added into HepG2 cells, and 5 μg/ml blasticidin was used to select stable transfectants 48 h post-transduction. The medium was changed every 3 to 4 days until Geneticin or blasticidin-resistant colonies appeared. Single colonies were picked and grown in selection medium in 24-well-plates.

### Cell viability assay

Cell viability was measured by MTT (3-(4,5-dimethylthiazol-2-yl)-2,5-diphenyl tetrazolium bromide) assay by solubilization the formazan with DMSO (dimethyl sulfoxide). Stable transfections of HepG2-CAV1, HepG2-CAV M1, HepG2-CAV M2, HepG2-GFP, HepG2-shRNACAV1 and vehicle control were seeded in 96-well plates at a density of 4000 cells/well. After overnight culture, the cell were treated with different final concentrations of RES (0, 10, 20, 30, 50, 100, 150, 200, 300 μmol/l). Control cultures containing absolute DMSO (0.1–0.3% dimethyl sulfoxide) were also established. Different RES concentrations were prepared freshly at each use by dissolving RES powder in absolute DMSO followed by serial dilutions in medium. Experiments were done in triplicates. Cell viability was measured by MTT assay at 24, 48 and 72 h culture time. The quantity of formazan product was measured by spectrophotometric microtiter plate reader (Dynatech Laboratories, Alexandria, VA) at 570 nm wavelength. Results were expressed as a percentage of growth, with 100% representing control cells treated with DMSO alone.

### Apoptosis and cell cycle distribution analysis

Cells were plated in 10-cm culture dishes and grown to 60–70% confluence within 24 hr. After overnight culture and cell adherence to the bottom, the culture medium was replaced by FBS-free DMEM. After 12 h, DMSO (0.1–0.3%) or RES (0–300 μmol/l) was added. Both adherent and floating cells were harvested 24 h, 48 h and 72 h after treatment. Subsequently, cells were fixed with 70% ethanol in ice-cold PBS and stained with propidium iodide (final concentration of 50 mg/L) in the dark for 30 min at room temperature. Finally, cells were subjected to apoptosis and cell cycle analysis by flow cytometry using a FACS Calibur. All experiments were performed in duplicate.

### RES treatment of the HepG2 xenografts in nude mice

The mice in this study were supplied by the Vital River Laboratory Animal Technology Co. Ltd. (SCXK (Beijing), 2007-0001), which is certified by the Charles River Laboratories (CRL, USA). All mice were cared for and maintained in accordance with animal welfare regulations under an approved protocol by the Beijing Bureau of Science Animal. 40 Balb/c-nu female nude mice weighing 17–20 g were randomly assigned to 5 groups. Xenografts were established by injecting 5 × 10^6 ^HepG2 cells with different stable transfectants (none, He-CAV1, He-CAVM1, He-CAVM2, He-GFP and He-CAVRNAi) in 200 μl PBS into the back of each mouse. Ten days after inoculation, mice were divided into a control group and a RES treatment group (each group including four mice; two CAVRNAi-transfected mice and one HepG2-transfected mouse died before RES administration). RES (15 mg/kg body) was administered intra-peritoneal once every other day for 21 consecutive days. Untreated HepG2-implanted mice were given sterilized water following the same schedule. Tumor volume was determined every 2–3 days by direct measurement with calipers and calculated using the formula, [width^2 ^(mm^2^) × length (mm)]/2. After scarification on day 30 tumor specimens and livers of each animal were removed, weighed and the RES content in both tissues was determined using HPLC analysis.

### Resveratrol analysis by HPLC

Cells were harvested in ice-cold PBS (1 mL per 50 cm^2 ^flask) and pelleted at 1500 × g for 5 minutes after washing them twice in ice-cold PBS. Cells were re-suspended in 50 μl NP-40 Cell Lysis Buffer (50 Mm Tris-HCl, 150 mM NaCl, 1% Nonide P-40, pH7.8) and homogenized by sonication for 10 seconds on ice. The protein concentration of cell lysates was determined by a bicinchoninic acid (BCA) kit analysis. An equal volume of 5.6 μg/ml inner standard solution was added (trans-Ferulic acid dissolved in methanol). The mixture was vortexed for 5 min, followed by centrifugation at 12,000 × *g *(4°C for 15 min). Twenty μl samples were injected into the HPLC device (Agilent 1100 series), separated on columns (Hypersil C18), eluted by mobile phase consisting of methanol:water: phosphate acid = 45:55:0.1 (v:v), at a flow rate of 0.8 mL/min, room temperature, and detected by Diode Array Detector at 320 nm. To test whether the molecular structure of RES is similar to diethylstilbestrol (DES), 10^-6^~10^-4 ^M/L DES plus RES were set to compete for ER activation. Furthermore, 10^-5 ^M/L Tamoxifen was added 4 h before RES administration.

### Synthesis of RES derivative fluorescent probes

RES derivatives were synthesized with the modification of dimethylaminonaphthalene sulfonyl chloride (dansyl chloride, DAN). After laser excitation of RES-DAN at 403.8 nm the emitted fluorescence of the RES derivates could be was measured at 530 nm and was assessed to compare the intra-cellular distribution of RES with that of CAV1. A solution of 228 mg (1.0 mmol) of RES in 10 mL of acetone was added into a mixture of 1 g of K_2_CO_3 _and 10 ml acetone in N_2 _atmosphere, 270 mg (1.0 mmol) of DAN in 10 mL of acetone added in sequential drops while cooling with an ice/water bath. The reaction mixture was stirred for 20 min at room temperature and heated to reflux for 2 h. The organic solution was filtered, dried by evaporation and allowed to crystallize in acetone to result in a yellow powder. Cells were then exposed to RES-DAN (300 μmol/L) for 2 h, after washing them twice with ice-cold PBS. The intra-cellular distribution of recombinant CAV1 was detected by incubation with mouse anti-GFP monoclonal antibody (1:200) and Cy3-conjugated goat anti-mouse antibody (1:500) for 45 min. After three additional washings, the co-localization of RES and CAV1 were observed and photographed using a Zeiss 510 laser confocal microscope [29].

### Confocal immunofluorescence imaging and immunohistochemistry

After incubation with or without 200 μmol/l RES for 24 h, cells were fixed with methanol/glacial acetic acid solution (3:1) for 15 min, permeabilized with 0.25% Triton+5% DMSO at 37°C for 20 min, blocked with TBST containing 5% defatted milk powder at 37°C for 2 h, incubated with rabbit anti-human CAV1 antibody and mouse anti-Topoisomerase-alpha (Ki-S1) (1:150) and blocked at 4°C overnight. Cells were then washed three times with TBST before and after incubation together with Alexa Fluor 488-conjugated goat anti-rabbit IgG (H+L) antibody and Cy3-conjugated goat anti-mouse antibody (1:500) for 45 min. The results were observed and photographed using a Zeiss 510 laser confocal microscope. The paraffin-embedded tumor samples were cut in-5 μm-thick sections with a microtome. After de-paraffinization, rehydration and antigen recovery, tissue sections were examined for expression of CAV1 and Topoisomerase-alpha proteins by CAV1 and Topoisomerase-alpha antibody. Primary antibody staining was followed by incubation with anti-mouse or anti-rabbit secondary IgG polymer conjugated with HRP or Alkaline phosphatase and signals were verified using Double Polymer Staining Detection System (ZSGB-BIO, China).

### Immunoblotting

Immunoblotting of phosphorylated ERK1/2, p38 kinase, and caspase-3 was carried out using phospho-specific MAP kinase antibodies against phosphorylated sites of ERK1/2, p38 kinase, or active caspase-3, respectively. As control, total ERK1/2, p38 kinase, and caspase-3 were analyzed with the respective specific antibodies following manufacturer's instructions (Santa Cruz Biotechnology). In brief, HepG2 cells or HepG2 Cells with different transfectants were starved for 24 h in 0.1% FBS DMEM at 37°C, in a 5% CO2 atmosphere incubator. Cells were then treated with RES (10–200 μmol/l) or DMSO (0.1%) for 24 h. In addition, another group of HepG2 cells treated with 20 μm SB202190 for 1 h followed by treatment with 200 μM RES were cultured for an additional 24 h. Cells were then washed once with ice-cold PBS and lysed in 200 μl lysis buffer (50 Mm Tris-HCl, 150 mM NaCl, 1% Nonide P-40, Ph 7.8) and protease inhibitor. After sonication and centrifugation (10,000 g for 15 min,) equal lysates (20 μg) were tested for levels of CAV1, phosphorylated ERKs, p38 kinase and caspase-3 levels by Western immunoblotting using specific antibodies and chemi-luminescence detection as previously described [30].

### Statistical analysis

All experiments were repeated three times. Data are presented as the mean ± SD. Statistical significance was evaluated by an ANOVA and a Bonferroni adjustment applied to the results of a *t*-test performed with SPSS software. Differences between groups were analyzed by a Student's *t*-test. *P *< 0.05 was considered statistically significant.

## Results

### Dose-and time-dependent cell death induced by RES in human hepatoblastoma carcinoma HepG2 cells

**Figure 1:**
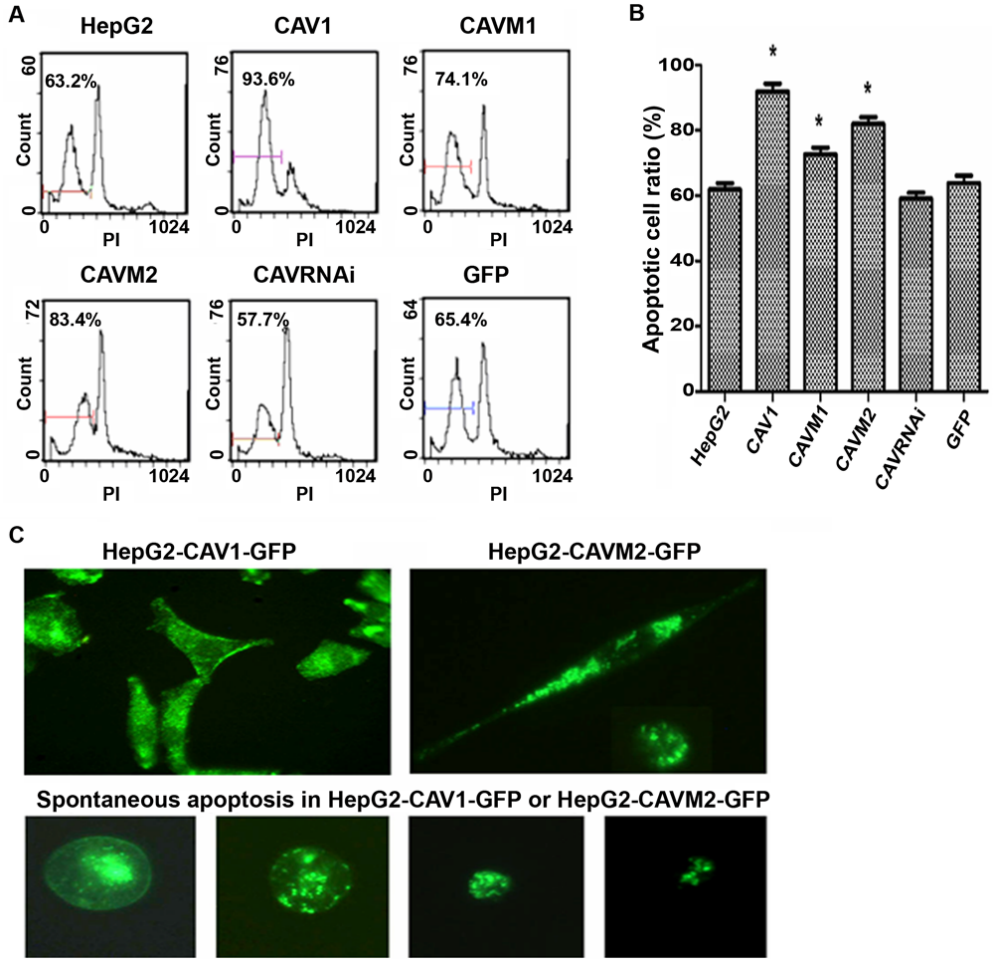
**(A) The six cell groups were pre-treated with 200 μM RES for 48 h, and apoptotic cell ratios were then measured by flow cytometry**. (B) Percentage of dead cells calculated for HepG2 cells variants treated with 200 μM RES for 48 h. Percentage of cell death was calculated over control. Data are presented as mean ± SD. Values represent the average of three different experiments. (C) Fluorescence imaging of CAV1 and CAVM2 overexpressing cells. *, statistical differences from the HepG2 cell control, p < 0.05.

To determine whether CAV1 is involved in the cytotoxic and pro-apoptotic activity of RES, HepG2 cells were treated with different doses of RES (0, 10, 30, 50, 100, 200 and 300 μmol/L). MTT and flow cytometry were used to detect inhibitory effects of RES on the growth of serum-stimulated HepG2 cells. As shown in Table 1 and 2 and Figure 1A and Figure 1B, the MTT assay indicates that RES inhibits significantly the growth of serum-stimulated HepG2 cells in a concentration-dependent manner. Cell cycle distribution indicated that high concentrations of RES induced a marked increase in cell number in sub-G1 and G0/G1 phase, with a corresponding decrease in other phases. Interestingly, concentrations of RES between 10 and 100 μM induced a modest but reproducible increase in cells at S phase. Increased apoptosis ratios were observed at increasing RES concentrations (Tables 3 and 4 and Figure 1). HepG2 cells were also treated with 200 μmol/L RES for 24, 48 and 72 h; cell growth inhibition increased in time in the control HepG2 cell lines from 55.45 ± 1.4, 68.91 ± 1.8, 78.83 ± 3.9 compared to baseline levels after 24, 48 and 72 h respectively. Significant increase of growth inhibition ratio was observed in HepG2 cells over-expressing CAV1 (68.32 ± 2.0, 80.12 ± 1.7, 90.02 ± 4.0, Table 2) and a significant reduction was observed in HepG2 cells in which CAV1 activity was inhibited (CAVRNAi). CAV1 and CAVM2 over-expressing HepG2 cells induced spontaneous apoptosis and increased the cytotoxic and pro-apoptotic effects of RES. CAV1 or CAVM2 promote apoptotic cell death by inducing plasma membrane crimple, small volume changes, increased density and changes in nuclear morphology (Figure 1C). A statistically significant difference (p < 0.05) was observed in apoptotic index at 50, 100, 200 and 300 μmol/L RES concentrations (10.93 ± 1.5, 31.2 ± 2.1, 63.2 ± 0.8, 80.6 ± 1.9) in CAV1 over-expressing cells (17.91 ± 2.5, 78.7 ± 1.7, 93.6 ± 2.0, 97.1 ± 1.7, Table 3). In contrast, apoptotic cells were significantly reduced in HepG2 cells expressing scaffolding domain deleted (CAV1^Δ^81–101) mutant. Down-regulation of CAV1 expression by shRNA correlated with decreased RES-induced growth inhibition (Table 3). These results suggest that RES can induce a dose- and time-dependent death of HepG2 cells, and over-expression of CAV1 can increase the cytotoxic and pro-apoptotic activity of RES even more.

**Table 1:** Cell growth inhibition of HepG2 cells by 24 h treatment with 0.1–0.3% DMSO, RES or 5-FU

Cell growth inhibition ratio (%)									
Cell groups		Res (μM)							5-FU (μM)
	DMSO	10	20	30	50	100	200	300	100
HepG2	0.046	11.88	17.49	22.03	30.50	45.95	51.45	65.93	50.86
CAV1	0.035	19.72	24.08*	37.13*	45.54*	57.54*	68.32*	87.89*	64.66*
CAVM1	15.97	22.63	25.03	33.82	45.98	55.78	72.34	48.48	0.047
CAVM2	13.91	19.39	26.44	34.77	47.55	57.27	76.56	42.46	0.029
RNAi	0.023	8.15	12.94	16.07*	24.34*	33.52*	41.23*	55.37*	32.83*
GFP	0.034	10.32	18.07	22.34	30.84	45.37	54.04	67.12	53.23
CAV1	0.035	19.72	24.08*	37.13*	45.54*	57.54*	68.32*	87.89*	64.66*

*P < 0.05 vs control, [ ± SD, SD = 0.8~2.5), n = 3]

RNAi = CAVRNAi

**Table 2:** Cell growth inhibition of HepG2 cells by 24, 48 and 72 h treatment with 200 μM RES

Cell growth inhibition ratios (%)			
Cell groups	time		
	24 h	48 h	72 h
HepG2	55.45 ± 1.4	68.91 ± 1.8	78.83 ± 3.9
DMSO	1.00 ± 0.9	1.53 ± 1.6	1.72 ± 0.7
CAV1	68.32 ± 2.0*	80.12 ± 1.7*	90.02 ± 4.0*
CAVM1	55.78 ± 1.0	74.83 ± 2.8	82.46 ± 1.6
CAVM2	57.27 ± 1.2	76.79 ± 1.6	84.35 ± 2.6
CAVRNAi	41.23 ± 1.5*	55.39 ± 1.2*	68.27 ± 1.9*
GFP	54.04 ± 1.6	70.06 ± 1.1	76.27 ± 1.7

*P < 0.05 vs control, ( ± SD, n = 3)

**Table 3:** Apoptosis induction in HepG2 cell variants by 48 h treatment with DMSO or 20–300 μM RES

	Percent Apoptosis (%)					
Cell groups		Res (μM)				
	DMSO	20	50	100	200	300
HepG2	1.53 ± 1.6	6.83 ± 1.9	10.93 ± 1.5	31.2 ± 2.1	63.2 ± 0.8	80.6 ± 1.9
CAV1	3.62 ± 1.8	13.2 ± 1.0	17.91 ± 2.5*	78.7 ± 1.7*	93.6 ± 2.0*	97.1 ± 1.7*
CAVM1	2.19 ± 1.8	9.62 ± 1.1	13.5 ± 1.8	23.1 ± 0.9	74.1 ± 1.8*	90.3 ± 0.6*
CAVM2	3.08 ± 1.3	11.5 ± 1.4	15.3 ± 1.6	50.1 ± 1.7*	83.4 ± 1.5*	93.5 ± 2.4*
CAVRNAi	1.37 ± 1.7	5.05 ± 1.4	9.78 ± 1.1	24.8 ± 2.5	57.7 ± 2.4	75.4 ± 3.1
GFP	1.44 ± 1.1	6.05 ± 1.8	11.2 ± 2.0	32.7 ± 1.6	65.4 ± 2.1	82.3 ± 3.0

*P < 0.05 vs control, ( ± SD, n = 3)

**Table 4:** Cell cycle distribution of HepG2 cells after treatment with or without RES for 48 h

	Cell cycle distribution														
	Res (μM)														
*Cell groups*	DMSO			20			50			100			200		
	G1	G2	S	G1	G2	S	G1	G2	S	G1	G2	S	G1	G2	S
HepG2	76.3	9.6	14.1	73.0	13.5	13.5	25.3	4.8	69.9*	34.9	7.4	57.7*	72.7*	8.8	18.5
CAV1	57.9	10.0	32.1	15.4	7.5	77.1*	27.5	21.4	51.1*	65.8*	1.6	32.6	73.5*	24.2	2.3
CAVM1	58.9	16.0	25.0	69.5	8.9	21.6	33.9	10.5	55.7*	39.9	20.0	40.1*	79*	8.6	12.4
CAVM2	68.2	11.1	20.7	72.5	3.8	23.7	30.5	8.4	61.1*	75.8*	3.1	21.1	74.1*	8.5	17.4
CAVRNAi	57.8	10.2	38.1	45.3	16.4	32.3	37.5	21.1	55.0*	64.9*	19.5	15.7	69.2*	13.1	17.7
**GFP**	77.4	5.9	16.7	73.2	14.0	12.5	22.3	6.0	71.7*	30.4	7.8	61.8*	70.0*	10.5	20.5

*P < 0.05 vs control, [ ± SD, SD = (0.8~3.7), n = 3]

### Synergistic anti-tumor activity of RES and CAV1 in nude mice

**Figure 2:**
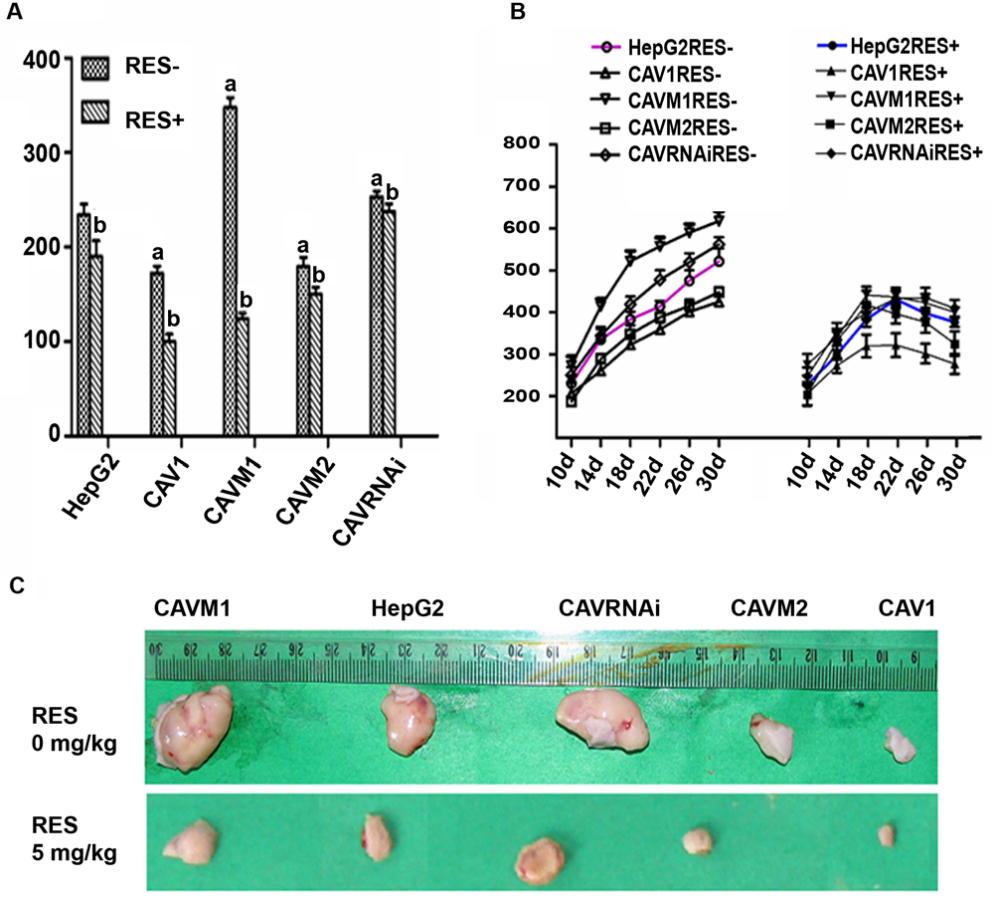
**Effects of RES treatment on final tumor weight (A) and volume (B, C) in HepG2 cell variant xenografts**. CAV1, CAVM1, CAVM2, CAVRNAi and HepG2 cells (5 × 10^6 ^cells/0.2 ml) were implanted subcutaneously into the back of Balb/c-nu female mice on day 0. RES treatment (15 mg/kg body) was started ten days after implantation. The tumor volume was calculated every 2 to 3 day. Values represent means × SEM, *n *= 4. a. *P *< 0.05 vs. control group. b. *P *< 0.05 vs. corresponding untreated group.

The above results indicate that CAV1 is a potentiator of the effects of RES on HepG2 cells *in vitro*. We next evaluated the activity of CAV1 mutants on the growth of HepG2 cells in nude mice subjected or not to RES treatment. HepG2 cells expressing the different CAV1 mutants (5 × 10^6 ^cells/animal) were implanted subcutaneously in the animals back. Within 30 days of implantation, GFP control vector HepG2 cells had an average tumor size of 400 ± 15 mm^3^. In contrast, xenografts from cells stably expressing CAV1 or CAVM2 were significantly smaller with an average tumor size of 325 ± 10 mm^3 ^and 340 ± 13.4 mm^3 ^(Figure 2 and Table 5). On the other hand the over-expression of mutant CAVM1 protein with deletion of the scaffolding domain 80-101aa promoted proliferation and malignant transformation compared to the parental cell lines and GFP vector-only transfectants (586 ± 21 mm^3^). RES (15 mg/kg body) administered intra-peritoneal every other day for 21 consecutive days starting at day 10 after tumor cell inoculation induced significant inhibition of tumor growth in all HepG2 cells whether wild type or expressing one of the various mutant constructs (Table 5). However, regression was more dominant in xenografts of HepG2 cells stably expressing CAV1. Furthermore, RES could reverse CAVM1 or CAVRNAi proliferative effects.

**Table 5:** Effect of RES on HepG2 variant xenograft weight

**Group**	**RES** **(mg/kg)**	**n**	**Tumor** **weight/mg**	**Inhibitory rate %**	**Intra-group inhibitory rate%**
HepG2	0	4	222.50 ± 22.5		
HepG2	15	4	173.33 ± 33.3^a^	22.11	22.11
CAV1	0	4	165.50 ± 10.2 ^a^	25.84	
CAV1	15	4	92.50 ± 15.1^b^	58.43	44.12
CAVM1	0	4	337.50 ± 20.6 ^a^	-51.68	
CAVM1	15	4	117.50 ± 12.5 ^b^	47.19	65.19
CAVM2	0	4	170.00 ± 18.9 ^a^	23.61	
CAVM2	15	4	142.50 ± 15.1 ^b^	35.96	16.17
CAVRNAi	0	2	247.50 ± 7.07 ^a^	-11.21	
CAVRNAi	15	4	230.00 ± 6.80^b^	-3.37	7.07

Values are means ± SEM, *n *= 4.

a. *P *< 0.05 vs control group.

b. *P *< 0.05 vs corresponding untreated group (RES at doses of 0 mg/kg)

### HPLC analysis of RES-treated cells

**Figure 3:**
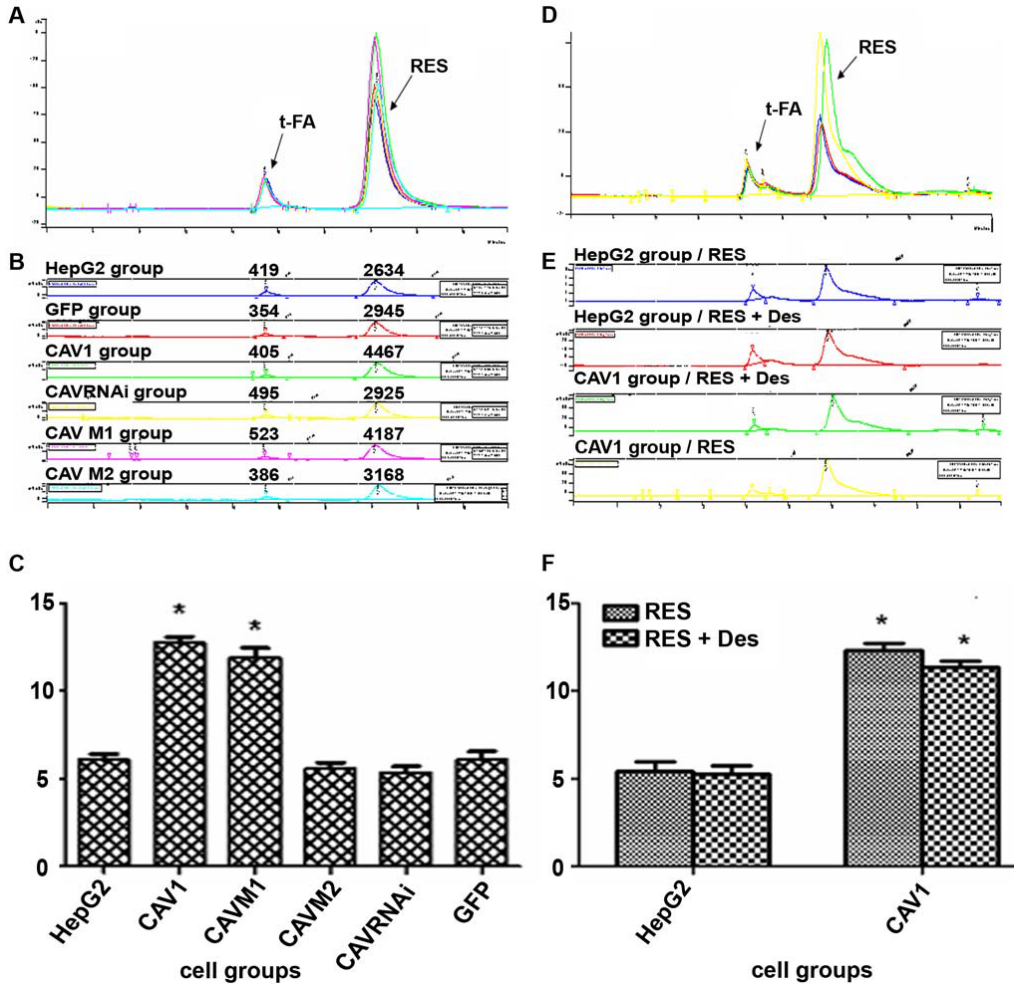
**(A) HepG2 variants were pre-treated for 24 h with 200 μM RES and RES concentrations were detected in the cytoplasm by HPLC**. (B) – Values for individual variants. (C) – Res concentration in the cytoplasm of individual HepG2 cells after 24 h pre-treatment with 200 μM RES. Each bar represents the mean ± S.E.M. of three independent experiments. (D) Cytoplasmic RES concentration in HepG2 variants after 10^-6^~10^-4 ^M/L Diethylstilbestrol (DES) plus RES measured by HPLC. (E) Individual variant values. (F) Mean ± S.E.M. values of three individual experiments. *, statistically significant differences between experimental variant and HepG2 cell control, p < 0.05.

After incubation with RES (50, 100, 150, 200, 250, 300 μM) for 2 h, 10 h, 24 h and 48 h, HepG2 cell plasma extracts were analyzed by HPLC. Intra-cellular RES concentration was increased in a dose- and time-dependent manner, but lower than the RES concentration in the supernatant (Data not shown). We therefore addressed whether CAV1 can induce endocytosis specifically and indeed intra-cellular RES concentration was increased about 2-fold in HepG2 cells stably expressing CAV1 or CAVM1 compared to HepG2 wild-type or GFP-transduced. Conversely, increased intra-cellular transport disappeared in cells stably expressing CAVM2 and CAVRNAi (Figure 3). To test whether the potential similar molecular structure of RES compared with DES may also display estrogen-like agonistic and antagonistic activity, we mixed 10^-6^~10^-4 ^M/L DES plus RES in a competitive assay. Intra-cellular RES concentration was not significantly different between the two conditions. Thus, RES concentration was increased two-fold in CAV1, CAVM1 HepG2 cells compared to HepG2 wild-type or GFP-transfectants independent of DES treatment (Figure 3D, E and Figure 3F). Furthermore, the estrogen receptor (ER) was blocked by 10^-5 ^M/L Tamoxifen citrate without altering the results (data not shown) suggesting that CAV1 induces endocytosis specifically and independent of ER activation. Furthermore, this data suggest that the 143–156 amino acids of the lipid-binding domain of CAV1 play a key role. On the contrary, the 81–101 amino acids scaffold-domain of CAV1 is irrelevant to CAV1-mediated internalization and trafficking of RES.

### Co-localization of RES and CAV1

**Figure 4:**
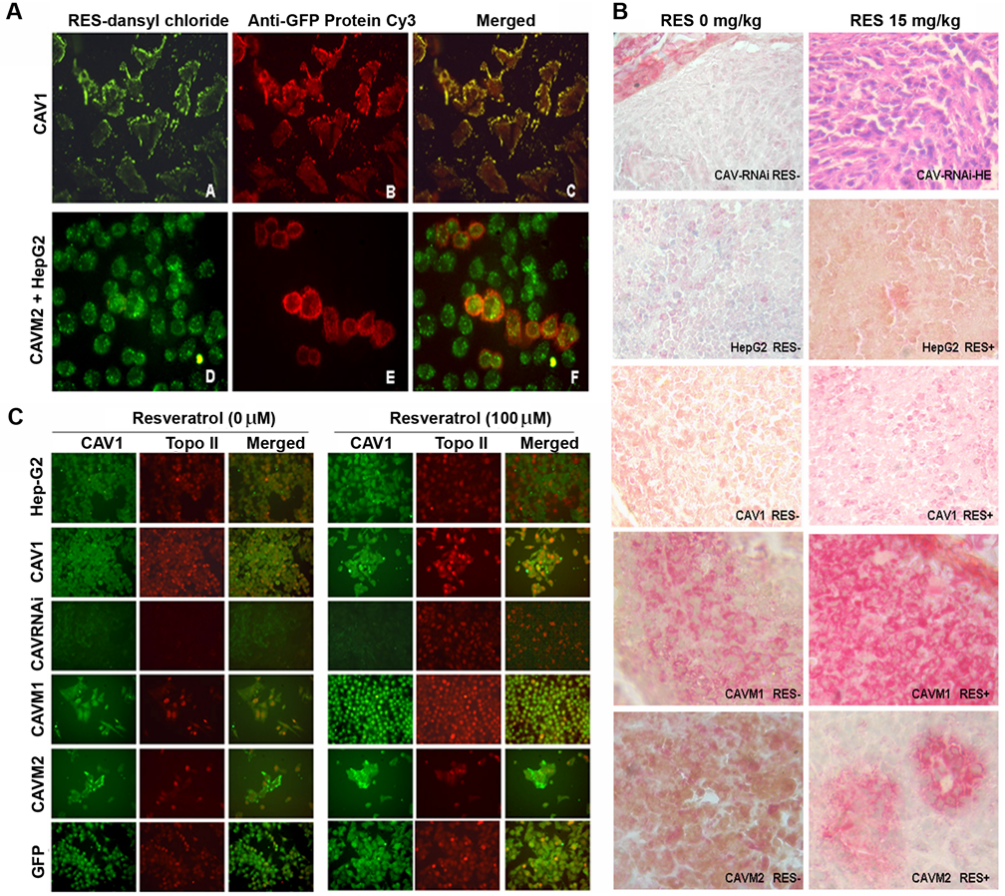
**(A) – Co-localization of RES and CAV1 in HepG2 cells – Transport of dansyl chloride-derived RES with green fluorescence (A) and recombinant CAV1 distribution with red fluorescence (B) and the two combination of both (C)**. Pooled HepG2 cells and CAVM2 cells bearing green (D) or red fluorescence (E). Over-expression of CAV1 by CAVM2 cell groups separates transfected from nontransfected HepG2 cells (E); the images are over-imposed in (F). Experiments were repeated 3 times, with similar results. (B) – Immunohistochemistry of tissue microarrays. (Magnification 400×). Immunoreactivity of CAV1 (red) and topoisomerase-alpha (Buffy) in HepG2 variant xenografts with (RES+) or without RES treatment (RES-). (C) Immunofluorescence of CAV1 and topoisomerase-alpha – Comparison of untreated (left) or RES-treated for 24 h (100 μM) (right). Localization of CAV1 and topoisomerase-alpha was visualized by indirect immunofluorescence. Microphotographs of a single field stained with anti-CAV1 (green) and topoisomerase-alpha (red) antibodies. (Magnification 400×) Experiments were repeated 3 times, with similar results.

To gather additional supporting evidence that RES may be transported into cells by CAV1 via its cholesterol shuttle domain, the co-localization of RES and CAV1 was investigated in HepG2 cells. Dansyl chloride-derived RES stained with green fluorescence (Figure 4A section A) and recombinant CAV1 staining with red fluorescence (Figure 4A section B) co-localized in the CAV1-expressing HepG2 (Figure 4A section C). We then analyzed the distribution of RES and CAVM2 (a cholesterol binding domain-defective CAV1 mutant) in pooled HepG2 cells and the over-expressing CAVM2 cells which displayed similar distribution of RES (Figure 4A section D). However, the over-expressing CAVM2 cells could be distinguished from non transfected HepG2 cells because of the red fluorescence (Figure 4A section E). In these cells, the labeling occurred mainly close to the membrane of the HepG2 cell and to a lesser extent in the cytoplasm where only a weak co-localization with RES could be observed (Figure 4A section F). These data strongly suggest that RES is transported into cells by CAV1.

### Confocal immunofluorescence and immunohistochemistry

CAV1 and topoisomerase-alpha protein expression in tissue and cells was studied by Immunofluorescence and Immunohistochemistry. As shown in Figure 4B and Figure 4C CAV1 and topoisomerase-alpha proteins were minimally expressed by HepG2 cells and respective xenografts not treated with RES. CAV1 was predominantly located around the cell membrane while topoisomerase-alpha was found in the nuclei. RES pre-treatment (100 μM) promoted the expression of CAV1 or topoisomerase-alpha while topoisomerase-alpha expression was inhibited completely in CAVRNAi cells. However, 100 μM RES pre-treatment recovered topoisomerase-alpha expression in CAVRNAi cells.

### RES increases CAV1 expression and MAPKs activity in HepG2 cells

**Figure 5:**
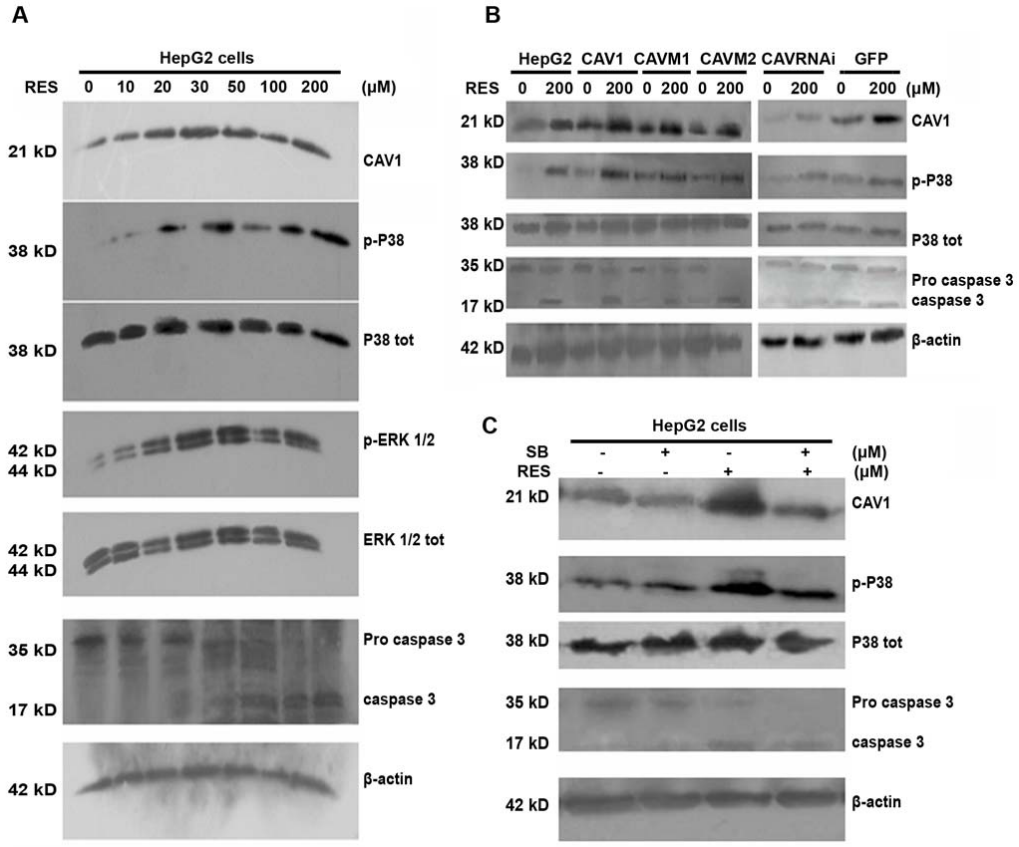
**(A) HepG2 cells were treated for 24 h with 0–200 μM RES; CAV1, MAPKs, and caspase-3 protein levels were further determined by Western blot**. (B) – HepG2 variants were treated with 200 μM RES for 24 h; CAV-1, caspase-3 and MAPKs protein levels were determined by Western blot. (C) – HepG2 variants were pre-treated for 30 minutes with or without 20 μM of the P38 inhibitor SB203580 prior to the 24 h treatment with 200 μM RES; CAV1, caspase-3 and MAPKs protein levels were determined by Western blot. Experiments were repeated 3 times, with similar results.

Previous studies showed that RES induces apoptosis through a caspase-dependent pathway. Therefore, the activity of caspase 3, a major component of the caspase pathways, was analyzed. In addition, the role ERKs and p38 kinase in regulation of caspase-3 -mediated apoptosis was studied by exposing cells to either DMSO (0.1–0.3%) or RES (0–200 μmol/l) for 24 h. CAV1, MAPKs, and caspase-3 protein levels were then determined by western blot. The data suggested that RES induces CAV1 expression in a dose-dependent manner from 30–50 μM and reached a peak value with higher concentration. Twenty-four hours after RES treatment, pro-caspase activity was reduced, and cleaved active caspase-3 was increased (Figure 5A). Phosphorylation of MAP kinases is essential for full kinase activation. Using phospho-specific antibodies against p38, ERKs and active caspase-3, we found that RES induced a rapid and prolonged activation of ERKs (10–50 μM) (4.1- fold induction compared to control), as well as activation of CAV1 or ERKs. Furthermore, RES increased, p-p38 and active caspase-3 expression (2.1-fold induction compared with control), whereas total ERKs and p38 kinase expression did not change. Similar results were detected in CAV1 expressing mutant cell lines (Figure 5B).

### Inhibition of p38MAP kinase leads to decreased apoptosis

As shown in Figure 1 and Table 1, RES treatment for 24 h induced apoptotic death in HepG2 cells. Activation of p38MAPK is involved in caspase-3-dependent cell death, but the role of p38 MAPK in RES-induced CAV1 expression and consequent apoptosis of HepG2 cells was not known. Therefore, HepG2 cells were pre-treated with the specific p38MAPK inhibitor SB203580 in presence or absence of RES and CAV1 and active caspase-3 expression were measured by Western blot. Indeed 20 μM SB203580 significantly reduced levels of RES-induced phospho-p38MAPK Resveratrol which was associated with significant differences in CAV1 protein expression and consequent apoptosis (Figure 5C).

## Discussion

Hepatocellular carcinoma (HCC) is the fifth most common cancer and accounts for more than 1 million deaths annually. The incidence of HCC in the Southeast Asia continues to rise steadily. Several systemic chemotherapies have been tested unsuccessfully against HCC, which remains incurable. Estrogen receptors (ERs) are localized to many sites within the cell, exposure to estrogens is a major known risk factor for breast cancer and other estrogen-mediated cancers. Experimental models suggest that estrogens stimulate hepatocyte proliferation *in vitro *and promote HCC growth *in vivo*. RES is a bioflavonoid that exists as *cis*- and *trans*-isomers, and the *trans*-isomer has greater anticancer and cardio-protective properties than the *cis*-isomer. As an estrogen analog activating ERα and ERβ, RES was suggested as a candidate chemo-preventive agent and a treatment option for HCC. CAV1, a member of Caveolin family may represent a tumor suppressor abolishing anchorage-independent growth of transformed cells and it is poorly expressed in HCC [31]. The close coupling between RES and CAV1 is suggested by ERα and ERβ co-localization within caveolin/lipid rafts and direct associations with caveolin-1 via its special scaffolding domain (amino acids 80 to 101). Therefore, we questioned whether RES interacting with CAV1 could suppress the proliferation of HCC. Preliminary experiments excluded the possibility that the CAV1-mediated activity of RES was due to direct CAV1-dependent activation of ERα and ERβ and proposed a novel mechanism responsible for RES-CAV1 mediated anti-cancer activity in HCC.

In this report, the data in HepG2 cells indicate that RES could inhibit the proliferation of HepG2 cells and increase their apoptosis in a time and dose-dependent fashion. In addition, our results are consistent with the notion that CAV or CAVM2 promote apoptotic cell death by inducing plasma membrane crimple, small volume changes, increased density, DNA fragmentation and changes in nuclear morphology. However, increased proliferation was not accompanied by a reduction in cell death in CAVM1 cells. An intriguing mechanism, in this regard, is the presence of scaffolding domain in caveolin-1 that binds to and inhibits the activity of several signaling proteins *in vitro *and *in situ*, including the EGF and Neu receptors, Src-family kinases (Src/Fyn), PKCs, eNOS and the heterotrimeric G-proteins [32]. Thus, it remains to be explained why over-expression of CAV1 by stable transfection enhances the anti-proliferative and pro-apoptotic effects of RES whereas knocking down CAV1 expression by RNAi technology induces the inverse result. Whether this observation reflects merely the superimposition of two tumor suppressor mechanisms, or CAV1 can interact synergistically with RES remains to be clarified.

Most chemo-therapeutic agents can traffic effectively to tumors and deliver their cytotoxic functions; however drug resistance is rapidly acquired predominantly through altered entrance of the drug inside cancer cells. This failure is due to rapid elimination by membrane proteins of intracellular anticancer agents pumped out of cells and cell organelles, decreasing intracellular concentrations and efficacy [33]. The HPLC data suggest that the distribution of RES is imbalanced between intra-cellular and extra-cellular compartments. Despite increased intracellular concentrations in a dose- and time-dependent manner, RES levels were always lower than in the supernatant (Data not shown). Interestingly, we found that intra-cellular RES concentration was increased 2-fold in HepG2 cells stably expressing CAV1 compared to HepG2 wild-type or GFP-transduced cells. To further explore the potential mechanism a scaffolding domain-defective CAV1 mutant (CAVM1) and a cholesterol shuttle domain-defective CAV1 mutant (CAVM2) were used to investigate the mechanisms of RES transport. CAVM1 transfected into HepG2 cells significantly elevated intracellular concentrations of RES up to 2 fold according to HPLC estimates; this was also consistent with CAV1 transfection experiments. However, CAVM2, with a non-functioning cholesterol shuttle domain did not enhance RES concentration in cells. More detailed characterization of CAV1-dependet RES transport required the synthesis of RES-dansyl chloride derivatives which could be utilized as fluorescent probes: RES was found to co-localize with CAV1 in HepG2 cells. In addition, RES endocytosis was not mediated through ERα and ERβ, as confirmed by lack of competitive inhibition by estrogens and tamoxifen.

Previous reports indicate that increasing levels of drug resistance are most likely due to decreased topoisomerase II protein levels [34]. In this study, we found that RES pre-treatment (100 μM) promotes the expression of CAV1 or topoisomerase-alpha while topoisomerase-alpha expression is inhibited completely in CAVRNAi cells. However, 100 μM RES pre-treatment recovered partially topoisomerase-alpha expression in CAVRNAi cells. These results displayed that reduced CAV1 protein levels might confer resistance and CAV1 may represent a new tool to avoid multi-drug resistance by cancer cells. Finally, we analyzed the relationship between RES and CAV1 expression and their role in inhibiting proliferation or inducing apoptosis of HepG2 cells. Immunoblotting analysis suggests that RES could up-regulate endogenous CAV1 expression, which further mediates the activation of the inhibitory p38MAPK cascade pathway and promotes the activation of the pre-apoptotic protein caspase-3.

Overall, this study confirms for the first time that over-expression of CAV1 enhances the transport of RES into HepG2 through its cholesterol shuttle domain rather than the scaffolding domain. This leads, in turn, in inhibition of proliferation and induction of HepG2 cell apoptosis mediated through the p38MAPK pathway and caspase-3 protein expression.

## Competing interests

The authors declare that they have no competing interests.

## Authors' contributions

HLY set up the protocols, HLY, WQC, XC, DLF, XL and YYX contributed to the experimental procedures and in the interpretation of the data, WYF, EW and FMM gave advises on the work and helped with the interpretation of the data, WYF, AW, EW and DFS supervised all the work and wrote the paper together with HLY and MFM. All authors read and approved the final manuscript.

## References

[B1] Jang M, Cai L, Udeani GO, Slowing KV, Thomas CF, Beecher CW (1997). Cancer chemopreventive activity of resveratrol, a natural product derived from grapes. Science.

[B2] Burkitt MJ, Duncan J (2000). Effects of trans-resveratrol on copper-dependent hydroxyl-radical formation and DNA damage: evidence for hydroxyl-radical scavenging and a novel, glutathione-sparing mechanism of action. Arch Biochem Biophys.

[B3] de la Lastra CA, Villegas I (2005). Resveratrol as an anti-inflammatory and anti-aging agent: mechanisms and clinical implications. Mol Nutr Food Res.

[B4] Palamara AT, Nencioni L, Aquilano K, De CG, Hernandez L, Cozzolino F (2005). Inhibition of influenza A virus replication by resveratrol. J Infect Dis.

[B5] Das DK, Maulik N (2006). Resveratrol in cardioprotection: a therapeutic promise of alternative medicine. Mol Interv.

[B6] Das S, Das DK (2007). Resveratrol: a therapeutic promise for cardiovascular diseases. Recent Patents Cardiovasc Drug Discov.

[B7] Ito T, Akao Y, Yi H, Ohguchi K, Matsumoto K, Tanaka T (2003). Antitumor effect of resveratrol oligomers against human cancer cell lines and the molecular mechanism of apoptosis induced by vaticanol C. Carcinogenesis.

[B8] Fulda S, Debatin KM (2004). Sensitization for tumor necrosis factor-related apoptosis-inducing ligand-induced apoptosis by the chemopreventive agent resveratrol. Cancer Res.

[B9] Zhang Q, Tang X, Lu QY, Zhang ZF, Brown J, Le AD (2005). Resveratrol inhibits hypoxia-induced accumulation of hypoxia-inducible factor-1alpha and VEGF expression in human tongue squamous cell carcinoma and hepatoma cells. Mol Cancer Ther.

[B10] Chan WK, Delucchi AB (2000). Resveratrol, a red wine constituent, is a mechanism-based inactivator of cytochrome P450 3A4. Life Sci.

[B11] Tseng SH, Lin SM, Chen JC, Su YH, Huang HY, Chen CK (2004). Resveratrol suppresses the angiogenesis and tumor growth of gliomas in rats. Clin Cancer Res.

[B12] Pozo-Guisado E, Merino JM, Mulero-Navarro S, Lorenzo-Benayas MJ, Centeno F, varez-Barrientos A (2005). Resveratrol-induced apoptosis in MCF-7 human breast cancer cells involves a caspase-independent mechanism with downregulation of Bcl-2 and NF-kappaB. Int J Cancer.

[B13] Szewczuk LM, Lee SH, Blair IA, Penning TM (2005). Viniferin formation by COX-1: evidence for radical intermediates during co-oxidation of resveratrol. J Nat Prod.

[B14] Tyagi A, Singh RP, Agarwal C, Siriwardana S, Sclafani RA, Agarwal R (2005). Resveratrol causes Cdc2-tyr15 phosphorylation via ATM/ATR-Chk1/2-Cdc25C pathway as a central mechanism for S phase arrest in human ovarian carcinoma Ovcar-3 cells. Carcinogenesis.

[B15] Bhat KP, Lantvit D, Christov K, Mehta RG, Moon RC, Pezzuto JM (2001). Estrogenic and antiestrogenic properties of resveratrol in mammary tumor models. Cancer Res.

[B16] Klinge CM, Blankenship KA, Risinger KE, Bhatnagar S, Noisin EL, Sumanasekera WK (2005). Resveratrol and estradiol rapidly activate MAPK signaling through estrogen receptors alpha and beta in endothelial cells. J Biol Chem.

[B17] Galluzzo P, Caiazza F, Moreno S, Marino M (2007). Role of ERbeta palmitoylation in the inhibition of human colon cancer cell proliferation. Endocr Relat Cancer.

[B18] Rejman J, Conese M, Hoekstra D (2006). Gene transfer by means of lipo- and polyplexes: role of clathrin and caveolae-mediated endocytosis. J Liposome Res.

[B19] Razani B, Schlegel A, Liu J, Lisanti MP (2001). Caveolin-1, a putative tumour suppressor gene. Biochem Soc Trans.

[B20] Ho CC, Huang PH, Huang HY, Chen YH, Yang PC, Hsu SM (2002). Up-regulated caveolin-1 accentuates the metastasis capability of lung adenocarcinoma by inducing filopodia formation. Am J Pathol.

[B21] Liu P, Rudick M, Anderson RG (2002). Multiple functions of caveolin-1. J Biol Chem.

[B22] Peterson TE, Guicciardi ME, Gulati R, Kleppe LS, Mueske CS, Mookadam M (2003). Caveolin-1 can regulate vascular smooth muscle cell fate by switching platelet-derived growth factor signaling from a proliferative to an apoptotic pathway. Arterioscler Thromb Vasc Biol.

[B23] Duxbury MS, Ito H, Ashley SW, Whang EE (2004). CEACAM6 cross-linking induces caveolin-1-dependent, Src-mediated focal adhesion kinase phosphorylation in BxPC3 pancreatic adenocarcinoma cells. J Biol Chem.

[B24] Graziani A, Bricko V, Carmignani M, Graier WF, Groschner K (2004). Cholesterol- and caveolin-rich membrane domains are essential for phospholipase A2-dependent EDHF formation. Cardiovasc Res.

[B25] Bowers JL, Tyulmenkov VV, Jernigan SC, Klinge CM (2000). Resveratrol acts as a mixed agonist/antagonist for estrogen receptors alpha and beta. Endocrinology.

[B26] Yang H, He S, Quan Z, Peng W, Yan B, Liu J (2007). Small interfering RNA-mediated caveolin-1 knockout on plasminogen activator inhibitor-1 expression in insulin-stimulated human vascular endothelial cells. Acta Biochim Biophys Sin (Shanghai).

[B27] Xu YY, Yang HL, Tu J (2006). The construction, Identification and primary functional analysis of pcDNA3.1/NT-GFP-Caveolin-1 and mutants plasmids. Chin J Atheroscler.

[B28] Yang HL, Jiang HJ, Fang WY, Xu YY, Liao DF, He FC (2005). High fidelity PCR with an off/on switch mediated by proofreading polymerases combining with phosphorothioate-modified primer. Biochem Biophys Res Commun.

[B29] Yang HL, Xu YY, DU LF, Liu CH, Zhao Q, Wei WJ (2008). Chemokine SR-PSOX/CXCL16 expression in peripheral blood of patients with acute coronary syndrome. Chin Med J (Engl).

[B30] Quan Z, Yang H, Yang Y, Yan B, Cao R, Wen G (2007). Construction and functional analysis of a lentiviral expression vector containing a scavenger receptor (SR-PSOX) that binds uniquely phosphatidylserine and oxidized lipoprotein. Acta Biochim Biophys Sin (Shanghai).

[B31] Yerian LM, Anders RA, Tretiakova M, Hart J (2004). Caveolin and thrombospondin expression during hepatocellular carcinogenesis. Am J Surg Pathol.

[B32] Torres VA, Tapia JC, Rodriguez DA, Parraga M, Lisboa P, Montoya M (2006). Caveolin-1 controls cell proliferation and cell death by suppressing expression of the inhibitor of apoptosis protein survivin. J Cell Sci.

[B33] Pakunlu RI, Wang Y, Tsao W, Pozharov V, Cook TJ, Minko T (2004). Enhancement of the efficacy of chemotherapy for lung cancer by simultaneous suppression of multidrug resistance and antiapoptotic cellular defense: novel multicomponent delivery system. Cancer Res.

[B34] Hazlehurst LA, Argilagos RF, Emmons M, Boulware D, Beam CA, Sullivan DM (2006). Cell adhesion to fibronectin (CAM-DR) influences acquired mitoxantrone resistance in U937 cells. Cancer Res.

